# Lactoferrin, a Natural Protein with Multiple Functions in Health and Disease

**DOI:** 10.3390/nu17213403

**Published:** 2025-10-29

**Authors:** Manuela Rizzi, Paolo Manzoni, Chiara Germano, Maria Florencia Quevedo, Pier Paolo Sainaghi

**Affiliations:** 1 Department of Health Sciences (DiSS), Università del Piemonte Orientale (UPO), 28100 Novara, Italy; 2 IRCAD (Interdisciplinary Research Center of Autoimmune Diseases), Università del Piemonte Orientale (UPO), 28100 Novara, Italy; 3 Department of Maternal-Infant Medicine, “Ospedale degli Infermi”, 13875 Ponderano, Italy; 4 Department of Public and Pediatric Health Sciences, School of Medicine, University of Turin, 10126 Turin, Italy; 5 Department of Translational Medicine (DiMeT), Università del Piemonte Orientale (UPO), 28100 Novara, Italy; 6 CAAD (Center for Autoimmune and Allergic Diseases), Università del Piemonte Orientale (UPO), 28100 Novara, Italy

**Keywords:** lactoferrin, antimicrobial activity, antiviral activity, immunomodulation, therapeutic applications, disease biomarker

## Abstract

Lactoferrin is a multifunctional glycoprotein showing multiple biological properties (antimicrobial, antiviral, antioxidant, antigenotoxic, prebiotic, probiotic) that play an essential role in maintaining host physiological homeostatic condition by exerting immunomodulatory and anti-inflammatory activities. Thanks to these biological properties, lactoferrin has widely been studied as a therapeutic agent in gastroenteric diseases, neonatal sepsis and necrotizing enterocolitis, lung diseases, and COVID-19, showing very heterogeneous results based on the disease considered and the population studied. Since lactoferrin is one of the main components of neutrophils’ secondary granules, it has also been investigated as a potential disease-monitoring biomarker, especially for diseases in which inflammation is a key component. This narrative review offers updated and comprehensive insights into the available literature on lactoferrin biology, biological properties, and clinical utility.

## 1. Introduction

Lactoferrin is a red-colored protein that was first isolated in bovine milk in 1939 by Sorensen and Sorensen. Its human counterpart was isolated only twenty years later, in 1960, by Johannson and two other independent research groups [1,2,3,4]. The characteristic color of this protein has been quickly explained by the discovery of the iron-binding ability of this molecule, determining its classification into the transferrin protein family [2,4,5,6].

As suggested by its name, lactoferrin is one of the major protein components of milk secretion in many mammal species [1,4,7,8]. Despite its common presence in mammalian milk, its levels are species-dependent as well as lactation stage-dependent. Several studies have quantified milk lactoferrin content, showing that human milk is the richest one, while its content in bovine milk is an order of magnitude lower. Moreover, it has been reported that lactoferrin abundance is maximal in colostrum, while it decreases in transitional and mature milk. Interestingly, it has also been observed that lactoferrin content in colostrum is higher in preterm delivers compared to women who had a natural delivery [3,4,9,10].

Currently, it is known that lactoferrin is not a milk-exclusive protein but has been described in various exocrine fluids, such as tears, saliva, bile, seminal fluid, and synovial fluid, with fluid-specific concentrations, as well as on the mucosal surfaces of the respiratory, urinary, genital, and intestinal systems [2,3,4,7]. Moreover, it has also been described in blood serum, where it is now recognized as one of the major components of neutrophils’ granules [4,6,8,9].

Breast milk represents the first nutrient for newborns and infants and is considered as a functional food, as it not only assures adequate nutritional support but it also beneficially affects the whole organism’s well-being. Considering that lactoferrin is one of the most abundant proteins in breast milk, it plays a positive role in sustaining the first stages of life, promoting tissue maturation and modulating immune response, thus assuring, together with the other milk components, the baby’s protection until the complete maturation of the immune system [11,12]. Moreover, its presence in other body districts highlights its involvement in several biological processes, making it a versatile player in host defense. Thanks to its multifaceted biological roles associated with high tolerability and safety profile, lactoferrin is commonly used not only as a nutritional supplement for humans of all ages but also as a food industry fortification agent and as a potential therapeutic agent [2,7,8,13,14,15].

**Figure 1:**
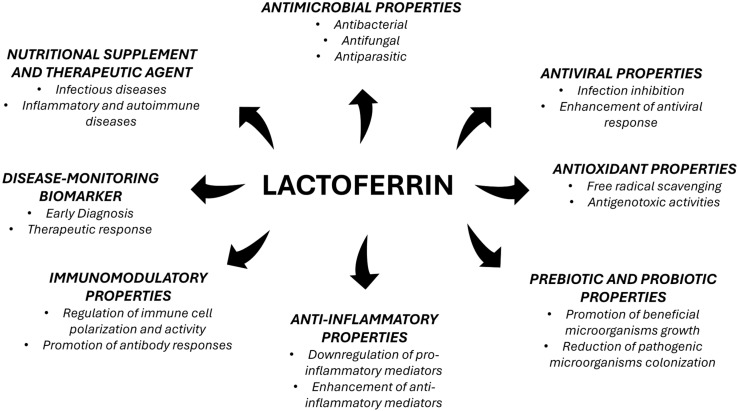
Summary of lactoferrin biological properties and clinical uses.

The aim of this narrative review is to summarize the available evidence about lactoferrin biology and the multifaceted roles of this protein in human health and disease, as shown in Figure 1.

## 2. Lactoferrin Structure

Lactoferrin is a cationic glycoprotein of the transferrin family with a molecular weight of about 80 kDa, with a highly conserved structure among different species [9,16]. From a three-dimensional structural perspective, the molecule is composed of an N-terminal and a C-terminal lobe linked by a helical sequence. Each one of the two globular lobes comprises two domains, with the iron-binding site located in the interdomain cleft [9,17,18,19].

From a physicochemical point of view, this protein shows a very high affinity for iron: each lactoferrin molecule could tightly bind two Fe^3+^ ions in a reversible manner, together with two synergistically bound CO_3_^2−^ ions, which seem to be essential in facilitating iron saturation [4,6,18,20]. Despite its well-known iron-binding ability, lactoferrin can also bind, albeit with lower affinity, several other positive metal ions, such as Co^3+^, Cu^2+^, Cr^3+^, Mn^3+^, Al^3+^, Zn^2+^, Ga^3+^, Cd^2+^, and Ni^2+^ [5,9,18].

Based on its iron-binding state, lactoferrin exists in three different biological forms: apolactoferrin, corresponding to the iron-free form (iron saturation < 5%); monolactoferrin, corresponding to the partially saturated form, which is prevalent in normal physiological conditions (native lactoferrin is characterized by a 15–20% iron saturation); and hololactoferrin, corresponding to the iron-bound form (iron saturation > 85%), which is prevalent in infected and inflamed sites. These forms show different characteristics, with the apo- form being more flexible and more susceptible to enzymatic degradation, while the holo- form, corresponding to a more compact structure, results in a more stable and proteolytically resistant form [2,9,18,21,22,23,24]. Interestingly, iron saturation level not only affects the physicochemical characteristics of the molecule but also some of its biological activities, with the apo- form showing stronger antibacterial and antioxidant activities than the holo- form. Moreover, it has been observed that the two existing iron-free isoforms showing no functional iron-binding (termed lactoferrin-β and lactoferrin-γ) exhibit RNase activity, while the iron-binding isoform (termed lactoferrin-α) does not [4,17,21,25].

Another notable physicochemical feature of lactoferrin is its positively charged surface, which is essential in facilitating lactoferrin binding to anionic biomolecules, such as DNA, heparin, and lipopolysaccharides. Furthermore, lactoferrin glycosylation, which occurs during protein maturation in the endoplasmic reticulum and Golgi apparatus, is mainly assured by N-linked mannose and N-acetylglucosammine residues: the number of glycosylation sites and the type of sugar residues is genetically regulated and affects both the whole molecule proteolytical susceptibility and thermal stability, as well as its immunogenicity [6,14,16,23,26,27].

## 3. Lactoferrin Digestion, Absorption, and Bioavailability

Similarly to other proteins of dietary origin, when reaching the stomach, lactoferrin undergoes a digestion process, finally leading to the production of smaller fragments. Interestingly, gastric digestion efficiency is different in adults and newborns, as the partially developed gastrointestinal system of the latter allows incomplete lactoferrin digestion, which could influence infants’ gut maturation [17,28,29,30,31]. Nevertheless, gastrointestinal digestion, albeit causing the loss of some functional properties of lactoferrin, could also be beneficial by generating new bioactive peptides, such as lactoferricin and lactoferrampin [2,6,8,16,32].

Considering that lactoferrin supplementation in humans is essentially based on bovine lactoferrin, it is not surprising that studies dealing with its fate in the gastroenteric system are focused on it. In particular, it has been reported that more than 60% of the orally administered bovine lactoferrin can pass the stomach, maintaining its structural integrity. The observed digestion rate has been linked to the gastric emptying rate and the buffering capacity of foods. On the other hand, bioactive peptides production upon digestion has been shown to greatly depend on intragastric pH, gastric emptying time, and enzymatic activities. Moreover, it should be remembered that the iron saturation of the molecule plays a key role in determining its breakdown, with the holo- form being more resistant to proteolysis [7,16,17,26,33]. Lactoferrin fate after ingestion has been investigated in several studies involving human subjects, allowing a deeper understanding of its gastrointestinal digestion. In a 2001 study, 12 healthy volunteers underwent nasogastric intubation for the administration of test drinks enriched with bovine lactoferrin (apolactoferrin in the presence or absence of a gastric pH buffer and hololactoferrin). The gastric survival of lactoferrin, as assessed by gel permeation chromatography, was more than 60% for all the test drinks, thus demonstrating that a substantial proportion of the orally administered lactoferrin could survive gastric transit, reaching in its intact form the small intestine [34]. Once the small intestine is reached, lactoferrin undergoes a further digestion step catalyzed by the enzymes present in the small intestine secretions. In a 2002 study, Troost and colleagues administered human recombinant lactoferrin-enriched beverages to eight female ileostomy patients and quantified lactoferrin excretion in the ileal effluent, showing that roughly all the ingested molecule was digested before reaching the colon [35]. Upon oral ingestion and gastric digestion, lactoferrin and its derivatives are absorbed by the intestinal mucosa and lymphatic system and then transported into the bloodstream, as demonstrated both in vitro and in vivo [17,30,33,36,37]. Moreover, as the amount of intact lactoferrin of dietary origin decreases while moving through the gastrointestinal system, it should also be considered that intact lactoferrin interacting with receptors in the distal part of the intestine could be of endogenous origin, as this biomolecule is recognized as one of the most important components of the secretory granules of neutrophils [4,6,8,9].

To exert its biological effects, lactoferrin needs to interact with specific receptors, which appear to be widely expressed in human tissues [30,38,39,40,41]. To date, no monospecific receptors for lactoferrin have been identified, as all the known receptors also bind other ligands. Among the known lactoferrin quasi-specific receptors are the intelectin 1 receptor, the low-density lipoprotein receptor-related protein 1, the heparan sulfate proteoglycans, the asialoglycoprotein receptor, and the Toll-like receptors 2 and 4, highlighting how different cellular effects depend on the signaling pathway activated. Moreover, it should be remembered that, when endocytosed, lactoferrin could reach the nucleus and act as a transcriptional activator, thus further expanding its ability to modulate biological responses in the human body [5,7,17,26,27,29,30,31].

As lactoferrin modifications occurring at the gastroenteric level affect its biological activities, different approaches to improve bovine lactoferrin oral bioavailability have been developed. Among those are the control of iron saturation, microencapsulation, PEGylation, and absorption enhancers. To begin with, the control of iron saturation is used to slow down the proteolytic degradation of bovine lactoferrin but is not effective in delivering the intact form of the protein to the intestine upon oral administration. On the other hand, microencapsulation is an effective method to protect bovine lactoferrin from enzymatic digestion. This approach is based on the formation of a protective structure formed by a protein- or polysaccharide-based shell or liposomes that, in addition to protecting the bovine lactoferrin core from degradation, could also allow a controlled release of the cargo. PEGylation, indeed, protects bovine lactoferrin from gastric degradation by increasing its steric hindrance, while the increase in total molecular mass inhibits its renal clearance. Finally, absorption enhancers are a group of chemicals able to increase the permeability or transport of the molecules to which they are conjugated across biological membranes. While all these modifications have proven to contribute to improved lactoferrin bioavailability, research is still needed to address their scalability in the pharmaceutical industry [28,32,33,42].

## 4. Lactoferrin Biological Actions

Based on its primary iron-binding activity, lactoferrin biological properties have been initially hypothesized to be related exclusively to that. To date, it is well accepted that even if several of its biological functions are strictly related to iron chelation, others are independent of it. As the knowledge about this multifaceted protein expanded, it appeared more and more clearer that lactoferrin plays a pivotal role in the whole organism’s well-being by exerting several protective actions, such as antimicrobial, antiviral, antioxidant, and anti-genotoxic activities, as well as anti-inflammatory and immunomodulatory actions. Furthermore, it is now well-known that lactoferrin also supports the whole organism’s homeostasis by exerting prebiotic and probiotic activities, thus supporting the proliferation of the beneficial commensal microflora while reducing the colonization ability of pathogenic strains.

The following sections briefly summarize the most important biological effects exerted by lactoferrin in the host and their contribution to the maintenance of a healthy status.

### 4.1. Antimicrobial Activity

In recent decades, evidence about a continuous increase in pathogen resistance to the commonly available antimicrobial treatments, as well as the emergence and re-emergence of epidemics, fostered the research of novel bioactive compounds aimed at fighting this enormous health challenge. Among the promising approaches that have been developed, the use of antimicrobial proteins and peptides has gained a growing interest.

Lactoferrin and its derived peptides (i.e., lactoferricin and lactoferrampin) have been proven to display a broad range of antimicrobial properties based on different mechanisms of action, such as iron sequestration, immunomodulation, and pathogen membrane disruption [7,43]. In particular, these natural compounds have been shown to be active against bacteria, fungi, and other parasites, thus representing a powerful tool in microbial infection management [7,24,43].

Among the antimicrobial activities of lactoferrin, the most studied one is represented by the antibacterial activity against both Gram-positive and Gram-negative strains (Table 1) [3,24,44,45,46].

From a mechanistic point of view, it has been observed that lactoferrin displays both bacteriostatic and bactericidal effects, as well as anti-biofilm and anti-adhesion properties [7,24,46,47].

The bacteriostatic effect of lactoferrin relies on its well-recognized iron binding ability, allowing an active reduction in iron availability for iron-dependent pathogens, thus contributing to host protection through so-called nutritional immunity [7,24,45,48]. On the other hand, the bactericidal effects mainly depend on its ability to specifically bind several pathogen membrane components, finally contributing to an increase in their susceptibility to the current antibiotic treatments. In particular, considering the Gram-negative strains, it has been demonstrated that lactoferrin is able to specifically bind to lipopolysaccharides moieties and porins, while, considering the Gram-positive strains, the binding mainly affects the lipoteichoic acid and/or teichoic acids moieties, finally leading to bacterial surface destabilization and increased permeability [7,45,46,48,49].

Furthermore, it has been observed that lactoferrin effectively prevents the bacterial biofilm formation at concentrations fivefold lower than that required for bacteriostatic effects. Lactoferrin’s ability to prevent bacterial biofilm formation has been demonstrated to be important in different clinical contexts, such as cystic fibrosis and periodontitis [7,46,50]. It is well-known that a major source of morbidity and mortality in cystic fibrosis patients is represented by *Pseudomonas aeruginosa* infection. This Gram-negative bacterium is one of the opportunistic pathogens that can colonize these patients’ airways, finally resulting in lung tissue destruction and eventually death. Experimental evidence showed that lactoferrin is able to inhibit bacteria adhesion and biofilm formation through the activation of a specialized motility form under iron-deprivation conditions. This has been elegantly demonstrated by time-lapse microscopy by Singh and colleagues who cultured *P. aeruginosa* in the absence or presence of lactoferrin. The researchers observed that, in optimal growth conditions (i.e., adequate iron availability in the medium), after bacterial division, the daughter cells remained near to the point of division, allowing microcolony formation, while in the presence of lactoferrin, which is known to chelate iron, thus reducing its availability for bacterial growth, daughter cells moved away from the point of division [7,46,50,51]. On the other hand, the major bacteria responsible for periodontitis is represented by *Porphyromonas gengivalis*, an anaerobic bacterium that has been reported to represent not only one of the primary bacteria responsible for tooth loss but also an important risk factor for cardiovascular as well as systemic disease development and progression. In this context, it has been demonstrated that lactoferrin is able to inhibit *P. gengivalis* aggregation into a biofilm community and inhibit the proteinase activity of its major virulence determinants in a time-dependent manner, while not directly affecting planktonic cells growth [7,49,52,53]. Interestingly, lactoferrin not only inhibits biofilm formation but also bacterial adhesion to host cells. As a matter of fact, it is known that many Gram-negative bacteria are characterized by specific appendages containing highly mannosylated glycans, called fimbriae or pili, that act like adhesins and mediate bacterial adhesion onto eukaryotic cells. Bovine lactoferrin has been shown to interact with these sugar residues on bacterial appendages, thus promoting bacterial aggregation instead of adhesion to the host cells and favoring their clearance from the infected district [54,55].

Lastly, bovine lactoferrin has also been proven to effectively reduce the motility of the flagellated enteropathogenic *Escherichia coli* strain as well as induce the degradation of specific virulence proteins thanks to its atypical serine protease activity [7,46,49,50].

Considering the antifungal and antiparasitic activities of lactoferrin and its derived peptides (alone or as lactoferrin chimera—the fusion product of lactoferricin and lactoferrampin), it has been hypothesized that the underlying mechanism of action is mainly related to pathogen surface destruction/destabilization, even if, especially in the case of the fungicidal activity, a role for iron deprivation cannot be excluded, thus supporting an additive and/or synergistic action of this molecule with commonly prescribed antifungal/antiparasitic drugs [24,46,47,56,57,58,59].

### 4.2. Antiviral Activity

Alongside wide antimicrobial activity, lactoferrin contributes to the whole organism’s protection thanks to its well-recognized antiviral activity. This biological activity of lactoferrin has largely been studied in the past and has gained new interest during the COVID-19 pandemic, when the lack of targeted and effective therapeutic interventions fostered the discovery of novel bioactive molecules as well as the repurposing of already known agents.

Since the last decade of the XX^th^ century, lactoferrin has been demonstrated to exert antiviral activity against several enveloped and naked viruses with RNA as well as DNA genomes (Table 2) [3,7,45,46,47,50,60,61,62]. This antiviral activity has been reported for both the whole protein and its derivatives, such as lactoferricines, albeit weaker than that of the native protein. Additionally, lactoferrin antiviral activity has been reported to be dependent on its origin, with bovine lactoferrin generally showing higher activity than its human counterpart [45,47,63].

From a general point of view, lactoferrin is known to exert its antiviral activity in the early phases of the infection process through different mechanisms, such as the inhibition of viral attachment and entry into host cells, the inhibition of viral replication and assembly, the inhibition of virus-driven cellular apoptosis, and the stimulation of immune responses [7,24,43,44,45,46,47,60].

Regarding the inhibition of viral attachment and entry, it has been demonstrated that a key role is played by host cell surface molecules, such as heparan sulfate, a glycosaminoglycan known to act as a viral receptor favoring virus entry in host cells. Thanks to its two glycosaminoglycan-binding domains located at the N-terminus of the molecule, lactoferrin is able to bind heparan sulfate moieties with high affinity on the host cell surface, thus efficiently blocking viral entry [7,46,50,63,64]. It is worth noting that not all lactoferrin-sensitive viruses require heparan sulfate as a receptor or co-receptor for host cell infection; as such, the protective action of such molecules also involves other mechanisms, such as the interaction with other host cell surface molecules, resulting in the reduction of viral endocytosis or the direct interaction between lactoferrin and the viral particle itself. In the last case, one of its targets is represented by hemagglutinin, a homotrimeric glycoprotein of the viral envelope playing a crucial role in viral adhesion and entry into host cells [7,45,46,50,63,65]. On the other hand, lactoferrin’s ability to interfere with viral particle replication and assembly has also been reported. In particular, it has been demonstrated that lactoferrin could not only efficiently block the nuclear export of viral nucleoproteins, as well as viral trafficking toward the nucleus, but also interfere with key viral enzymes involved in both viral infection and replication processes inside host cells. Moreover, lactoferrin could suppress viral replication through interferon activity induction [7,45,61,64,66,67].

Furthermore, lactoferrin has been proven to prevent viral spread by protecting the infected cells from virus-induced apoptosis by targeting caspase-3, a cysteinyl protease that represents one of the key regulators of the apoptotic process initiated by viral infection [45,68,69]. Finally, lactoferrin also protects the host from the damage of viral infection by acting on immune responses, especially by downregulating pro-inflammatory cytokines and chemokines expression and by stimulating immune cell activity, thus promoting viral clearance [60,62,66,68].

### 4.3. Antioxidant and Antigenotoxic Protection

Despite being an essential nutrient for cell growth, when present in abnormally high concentrations, iron could become toxic, leading to the generation of free radicals. Free iron toxicity depends on its ability to donate or receive electrons from adjacent molecules, damaging cellular structures and ultimately leading to the generation of noxious reactive oxygen species (ROS). Due to its natural ability to chelate iron, lactoferrin plays an important role in maintaining redox homeostasis by balancing ROS production and elimination rates through iron sequestration [32,70,71].

Lactoferrin antioxidant potential, both in terms of direct reduction in intracellular ROS and enhancement of antioxidant endogenous defenses, has been studied both in vitro and in vivo, with promising results supporting the potential protective role of lactoferrin consumption against oxidative stress-related damage to both cells and tissues [1,32,70]. As an example, from an in vitro point of view, Park’s research group demonstrated lactoferrin ability to decrease intracellular ROS levels and consequently suppress senescence and apoptosis in a human mesenchymal stem cell model by inhibiting Akt and caspase-3 signaling pathways [72], while Liu and coworkers demonstrated, in an enterocyte-like model, lactoferrin’s ability to reduce both intracellular ROS and malondialdehyde levels while increasing antioxidant defenses, such as glutathione peroxidase activity and nuclear factor erythroid 2-related factor 2 (Nrf2) protein expression [43]. Furthermore, Bodur and colleagues demonstrated that lactoferrin was able to significantly reduce lipid peroxidation and increase glutathione reductase, catalase, and superoxide dismutase in HepG2 cells treated with acrylamide [73]. Interestingly, lactoferrin’s ability to induce antioxidant enzymes is not solely found in its natural isoforms but is also retained by the recombinant human isoform obtained with a CHO expression platform [74], thus suggesting that, upon overcoming the still-existing barriers for its clinical use, recombinant human lactoferrin could also represent a promising therapeutic option. On the other hand, from an in vivo point of view, Okazaki and colleagues demonstrated in a rat model that bovine lactoferrin administration could effectively act as an antioxidant defense in a ferric nitrilotriacetate-induced renal tubular oxidative injury [75], while Al Zharani’s group showed, in a murine model, that oral lactoferrin supplementation could effectively ameliorate hematological and biochemical parameters in mercuric chloride-induced hepatic and renal oxidative stress [76]. Finally, Carvalho and colleagues reported lactoferrin ability to restore antioxidant defenses in a murine neonatal hypoxia–ischemia model through the enhancement of superoxide dismutase and glutathione peroxidase activity, the increase in reduced glutathione tissue levels, and the upregulation of Nrf2 and uncoupling protein 2 (UCP2) [77].

Given that one of the most dramatic consequences of the imbalance between ROS and antioxidant defenses is represented by oxidative DNA damage [71], it is not surprising that lactoferrin’s protective role has also been investigated in this context. In 2013, Habib and coworkers demonstrated, in an in vitro model, that lactoferrin was able to reduce DNA fragmentation induced by UV-photolysis in the presence of hydrogen peroxide and FeSO_4_, hypothesizing a protective action based on its ability to bind iron, thus inhibiting the Haber–Weiss reaction [78]. Similar results were obtained the following year by Ogasawara and colleagues, who demonstrated, in an in vitro model, lactoferrin’s ability to prevent DNA fragmentation due to ultraviolet irradiation in the presence of hydrogen peroxide. Interestingly, they observed that, in this specific experimental setting, lactoferrin-mediated protection was independent of its iron-chelating ability, as the molecule itself underwent degradation and partial aggregation upon stimulation, thus acting as a sacrificial ROS scavenger, directly interacting with hydrogen radicals to protect cellular DNA from oxidative injury [79]. More recently, lactoferrin’s protective effect against mycotoxin-induced DNA damage was also demonstrated in different cell lines, thus further supporting lactoferrin’s role in assuring human well-being [80].

### 4.4. Anti-Inflammatory and Immunomodulatory Functions

In addition to the well-known antimicrobial, antiviral, and antioxidant properties, lactoferrin also exerts important anti-inflammatory and immunomodulatory functions that play a key role in maintaining the whole organism’s well-being and are responsible for its wide use as an over-the-counter nutritional supplement [81]. Lactoferrin’s involvement in immune defense is based on its ability to activate innate and adaptive responses as well as to modulate cytokine and chemokine production and release, finely regulating inflammatory responses [1,28].

One of the simpler mechanisms by which lactoferrin takes part into immune protection is represented by its participation in innate defense mechanisms, especially thanks to its ability to interact with secreted IgA and defensins as well as to act as an opsonin, thus promoting invading agents’ clearance by phagocytosis [28,82].

Interestingly, lactoferrin involvement in immune system functioning is more complex, as this molecule is also a critical mediator of innate and adaptive responses, resulting in local and systemic effects aimed to maintain the whole organism’s homeostasis.

Lactoferrin immunomodulatory function is strictly related to its antimicrobial properties. As a matter of fact, it not only directly counteracts pathogen invasion but also interferes with the pathogen-associated microbial patterns (PAMPs)/toll-like receptors (TLRs) axis [1,82,83]. Moreover, lactoferrin could directly interact with immune cells by binding its specific receptors on their surface, thus modulating their proliferation, differentiation, and activation, as confirmed by different in vitro studies [70,82,84].

Lactoferrin’s immunomodulatory mechanism has been shown to depend on the immune cell considered. Regarding T lymphocytes, it has been observed that lactoferrin supports their proliferation and subsequent differentiation by acting as an alternative iron donor. Furthermore, it has been reported to affect the Th1/Th2 balance, thus orientating T cell responses towards the Th1 type [5,8,82,85,86]. Lactoferrin has also been shown to promote B cell differentiation and the subsequent antibody response [1,8,86,87]. Finally, lactoferrin is able to promote the recruitment and activation of antigen-presenting cells, thus bridging innate and adaptive immune responses [1,86,87,88].

Lactoferrin influences immune responses not only at the cellular level but also at the molecular level by modulating the production and secretion of soluble mediators, such as cytokines and chemokines, thus regulating immune cell functions and inflammatory responses. In particular, it has been observed that lactoferrin downregulates the most relevant pro-inflammatory cytokines’ (e.g., IL1β, IL6, and TNFα) expression, while stimulating the release of the anti-inflammatory ones, such as IL4 and IL10 [5,13,28,89]. The observed ability of lactoferrin to regulate immune mediators has been related to its ability to interfere with the NF-κB signaling pathway [1,90,91].

Additionally, lactoferrin has been reported to influence inflammatory responses in different ways. On the one hand, it has been reported to sustain macrophages’ polarization toward the tolerogenic phenotype [92], thus further supporting its role in maintaining immune homeostasis. On the other hand, lactoferrin, as well as its digestion-derived peptides, has been shown to reduce complement activation following inflammation, thus reducing the risk of damage to inflamed tissues [5,90,93,94].

### 4.5. Microbiota Diversity Promotion and Protection of Epithelial Barrier Integrity

In healthy humans, there are approximately 10^14^ commensal microorganisms living symbiotically within the intestinal tract, where they play a crucial role in ensuring the whole organism’s well-being, accounting for vitamin synthesis, fiber fermentation, xenobiotics metabolism, and preventing the overproliferation of pathological strains coming from the exterior [43,95,96]. Importantly, the intestinal epithelium not only acts as a physical barrier but also plays a direct role in maintaining microbiota homeostasis by secreting antibacterial compounds (e.g., definsins, cathelicidins, RNases). Mucosal immunity is also improved by the resident innate immune effectors (e.g., lymphocytes and lamina-propria plasmacytoid cells), which cooperate with adaptative immune system components (e.g., dendritic cells, T and B cells) to ensure whole-body protection from external noxious threats [43,97].

Lactoferrin is known to act both as a prebiotic and probiotic compound, and several researchers demonstrated its ability to sustain the proliferation of selected probiotic strains, such as *Bifidobateria* and *Lactobacillus*, over pathogenic ones, thus contributing to microbiota diversity [13,23,44,95,98,99]. Interestingly, some research groups proved that, in the intestinal lumen, lactoferrin’s biological actions target not only microbiota but also the epithelial layer supporting the commensal bacteria community, demonstrating the active modulation of enterocyte growth and maturation by lactoferrin [43,66,83,100].

Considering that damage to the epithelial barrier can result in the translocation of intestinal microorganisms and their metabolites into the bloodstream, thus resulting in the onset of several diseases, the growing interest in the lactoferrin–epithelial barrier relationship is not surprising. Recently, different authors reported lactoferrin’s ability to regulate the expression of several tight junction proteins, such as zonula occludens-1, claudin-1, and occludin [43,66,100,101], thus contributing to the maintenance of epithelial barrier integrity.

In addition to the gut, the female vagina is another body district characterized by the presence of a commensal symbiotic flora. In healthy women, vaginal microbiota is dominated by *Lactobacillus* strains, which protect the host from potential pathogens in different ways (i.e., by producing lactic acid, enhancing local immune responses, and producing antimicrobial compounds). On the other hand, vaginal dysbiosis is characterized by a reduction in resident lactobacilli (<10^6^ CFU/mL) associated with an increase in opportunistic pathogenic strains, mainly originating from the gastrointestinal tract [102,103,104].

Lactoferrin’s effectiveness in protecting the vaginal environment has been tested in vitro and in animal models as well as in some clinical trials. The obtained results suggest that lactoferrin, thanks to its prebiotic and probiotic, as well as antimicrobial, activities, is able to improve local microbiota, supporting lactobacilli proliferation over the pathogenic ones, thus helping the host to maintain the acidic environment essential to prevent bacterial as well as viral infections [23,28,103,104,105]. Furthermore, lactoferrin treatment could represent a promising solution for the treatment of recurrent infections by antibiotic-resistant pathogens, as it avoids the elimination of the beneficial flora usually induced by the classical antimicrobial therapy while inhibiting the opportunistic microorganisms’ spread and proliferation [106,107]. Finally, the known lactoferrin anti-inflammatory activity has also been proven to reduce IL-6 levels in cervicovaginal fluid, thus contributing to vaginal infection resolution and obstetrical complication reduction [28,103,104,105,108].

## 5. Lactoferrin Therapeutic Use

Based on its multifaceted biological actions, lactoferrin has been largely investigated in terms of therapeutic applications in different clinical settings. Lactoferrin therapeutic effects have been investigated both in pediatric and adult populations. While lactoferrin appears to generally exert a protective effect in pediatric patients against different diseases, the results of the studies focused on the adult population are often inconclusive, with some researchers reporting positive results in specific clinical settings, while others failed to obtain any clinically significant result. Moreover, well-designed clinical trials are limited.

The use of lactoferrin as a therapeutic compound mainly relies on the bovine equivalent, which is known to display a high sequence homology with the human protein and is easily available at a low cost [2,28,109]. Bovine lactoferrin’s safety for therapeutic use is well-recognized, as certified by the GRAS (generally recognized as safe) label granted by the US Food and Drug Administration in 2001 and by the recognition as a novel food ingredient by the European Food Safety Authority in 2012 [110,111].

The following sections summarize the available evidence about lactoferrin’s therapeutic effectiveness toward different diseases, such as gastroenteric diseases, neonatal sepsis, and necrotizing enterocolitis, lung diseases, and COVID-19.

### 5.1. Gastroenteric Diseases

Due to its well-known antimicrobial, anti-inflammatory, and iron-chelating abilities, lactoferrin use has gained growing interest in the field of gastroenteric diseases, both as a preventive and supportive therapeutic intervention. Such interest rises from some key characteristics of lactoferrin. Firstly, this molecule is known to act as a bactericidal and bacteriostatic agent toward many Gram-positive and Gram-negative bacteria involved in gastroenteric disease pathogenesis (e.g., *Enterococcus*, *Escherichia*, *Enterobacter*, *Helicobacter*, *Vibrio*), thus helping in controlling bacterial infections. Secondly, it has been reported to act as a prebiotic compound, thus promoting the growth of specific probiotic strains and helping to reduce antibiotic-dependent gastrointestinal side effects and intestinal dysbiosis. Thirdly, lactoferrin has been proven to exert a barrier-stabilizing effect in both in vitro and in vivo models of intestinal barrier damage by restoring tight junction morphometry and reducing epithelial apoptosis. Lastly, it is well-known that lactoferrin is able to reduce pro-inflammatory marker (e.g., IL1, IL6, IL8, TNFα) expression, thus contributing to inflammation resolution and tissue damage recovery, as demonstrated in vitro, where lactoferrin administration has proven to be able to upregulate intestinal stem cells (Lgr5) and proliferation (Wnt/β-catenin) markers, and in vivo, where it successfully alleviated aflatoxin M1-induced intestinal barrier dysfunction [22,44,54,109,112,113,114].

Among the gastroenteric conditions for which lactoferrin has been successfully used as a therapeutic approach, there is *Helicobacter pylori* infection, which represents a major pathogenic factor for chronic gastritis and peptic ulcers [115,116,117,118]. The interest in lactoferrin’s potential therapeutic value in this context dates back to the end of the previous century, when the first studies evaluated its in vitro and in vivo effectiveness against *H. pylori* commercial strains and clinical isolates [55,118,119,120]. These data have since been confirmed using both in vitro and in vivo models based on antibiotic-resistant strains [115,121,122,123]. Since the uncontrolled use and abuse of antibiotics worldwide is the major cause of the emergence of antibiotic-resistant bacterial strains, thus strongly reducing year by year the successful eradication rate of this infection, the actual clinical approach to this condition is represented by triple or even quadruple therapies based on a mix of antibiotics and proton pump inhibitors [115,121,123,124]. Despite being effective, due to the considerable use of antibiotics, these approaches are associated with gastrointestinal side effects mainly related to the destruction of the resident microbial flora and the generation of a pro-inflammatory environment [116,119,122,125]. In this context, it is not surprising that several researchers focused their work on the discovery of novel interventions to eradicate *H. pylori* infection or, at least, reduce its antibiotic resistance. The positive preclinical results obtained using lactoferrin thus fostered the design of clinical trials aimed at investigating its effectiveness as an add-on to the standard triple/quadruple therapy (Table 3). As of now, some clinical trials, but not all, have demonstrated that lactoferrin’s addition to the proton pump inhibitor/antibiotic mix increased the successful eradication rate of the classical therapy by taking advantage of the different lactoferrin antibacterial mechanisms of action compared to antibiotics, thus maximizing the eradication effect. Moreover, lactoferrin supplementation (especially in combination with probiotics) has been proven to reduce the common side effects associated with classical eradication schedule, thus further supporting its use in managing *H. pylori* infection [115,117,121,124,125,126].

As observed for *H. pylori* infection, lactoferrin’s antimicrobial properties are also gaining interest in the context of inflammatory bowel disease (IBD), a chronic inflammatory and relapsing disorder encompassing conditions such as Crohn’s disease and ulcerative colitis, whose incidence and prevalence is continuously increasing, regardless of sex and socio-economic conditions [2,41,128]. In addition to the antibacterial properties, there are other lactoferrin characteristics that are fostering its investigation as a potential therapeutic approach for both prevention and treatment of this pathological condition. Considering that IBD is characterized by an increased intestinal permeability, mainly due to a decrease in tight junctions’ functionality, the available in vitro data showing bovine lactoferrin’s ability to restore tight junctions’ morphology and function suggests a potential protective effect of this molecule [2,43]. Furthermore, considering that IBD is a clinical condition characterized by a pro-inflammatory milieu, lactoferrin-dependent downregulation of inflammatory cytokines, such as IL6, IL8, and TNFα, as demonstrated both in in vitro and ex vivo models, could represent a beneficial approach in disease management [2,54]. Finally, the known lactoferrin ability to boost mucosal immune responses and to promote macrophage shift from inflammatory to tolerogenic phenotypes could also represent a promising therapeutic approach for inflammatory bowel disease [2,43]. Interestingly, despite the encouraging results coming from in vitro and ex vivo models, as well as from animal studies, to date there are no controlled clinical trials available to evaluate lactoferrin effectiveness for IBD treatment. At the time of writing, the only clinical evidence regarding lactoferrin use in IBD management is represented by a case report showing a long clinical (at least 3.5 years) and symptomatic (4.5 years) remission in a Crohn’s colitis patient supplemented daily with bovine lactoferrin [129].

In conclusion, existing evidence highlights a promising role of lactoferrin in supporting gastroenteric disease management thanks to its ability to contrast microbiome dysbiosis as well as to promote anti-inflammatory responses through its immunomodulation activity. Despite in vitro and in vivo data availability, to define clear clinical guidelines in this field, well-designed clinical trials are needed to definitively confirm or disclaim its efficacy, as the available ones are mainly based on small patient cohorts, thus limiting the generalizability of the obtained results. Furthermore, especially regarding the field of IBD, the investigation of lactoferrin effectiveness is still at the beginning, with several promising preliminary results that could represent the starting point for the development of well-structured clinical trials aimed to shed light on the therapeutic and/or supportive role of lactoferrin supplementation in the clinical management of this disease.

### 5.2. Neonatal Sepsis and Necrotizing Enterocolitis

Sepsis and necrotizing enterocolitis are two severe conditions in newborns and especially in preterm infants, representing a critical health issue for these specific populations. Despite the development of potent therapeutic interventions, both the mortality and morbidity associated with these clinical conditions remain high worldwide, fostering the continuous search for effective therapies able to overcome the increasing emergence of antibiotic-resistant strains [3,130].

Considering its well-known antimicrobial and anti-inflammatory activities, as well as its role in supporting infants’ immune and gastrointestinal systems maturation, lactoferrin has been widely investigated as a potential well-tolerated and safe intervention for preventing and/or treating these potentially lethal conditions [3,83,130,131,132].

To date, several clinical trials have investigated lactoferrin (both bovine and human recombinant forms) enteral supplementation (alone or in association with probiotics) effectiveness in the treatment of neonatal sepsis and necrotizing enterocolitis, as summarized in Table 4.

Although some studies showed promising results, especially in terms of all causes of sepsis reduction [83,130,136,143], others showed inconclusive results [140,141,142]. Of note, positive results have been observed in the most vulnerable populations of very-low-birth-weight neonates and preterm ones, thus further supporting the beneficial effect of lactoferrin prophylaxis in early life [133,134,135,136,138,139,144,145].

Interestingly, despite the existence of human recombinant lactoferrin forms, such as talactoferrin, this formulation was used only in very few studies [138,139], while most of the available clinical trials were designed using bovine lactoferrin. This choice is mainly due to the higher production costs of human recombinant proteins and to the potential allergenic risk associated with the non-human glycosylation patterns of the recombinant forms [60,132]. Considering its high degree of homology with its human counterpart and the comparable biological activity, the use of the cheaper and more commercially available bovine form appears to be reasonable, especially considering the lack of adverse effects or intolerance manifestations described in all the bovine lactoferrin-based clinical trials [83].

In conclusion, the existing heterogeneity between the currently available evidence impairs the definition of clear guidelines for clinical intervention but supports the need to start lactoferrin prophylaxis as soon as possible, while it remains unclear which is the best intervention duration to ensure optimal results [130], thus supporting the continuous research on this topic with the aim to define the optimal therapeutic schedule to ensure an effective improvement in patients’ clinical condition.

### 5.3. Lung Diseases

Among the diseases affecting the lungs and respiratory system, the degree of severity varies, ranging from the mild ones, such as the common cold, to the most severe ones, such as acute respiratory distress syndrome. Overall, to date, lung diseases still represent one of the most important causes of hospitalization, disability, and death worldwide [146]. According to that, and considering that several of the existing therapies for their severe clinical manifestations are often associated with side effects, it is not surprising that the search for new compounds with therapeutic activities or able to act as add-ons to the common therapies is constantly advancing. Considering its well-known antimicrobial, antiviral, and immunomodulatory profile, lactoferrin has quickly gained interest even in this field.

Among lung diseases, respiratory tract infections represent a great healthcare burden, often associated with an excessive and/or uncontrolled use of antibiotics, finally leading to the development of antibiotic resistance in their pathogenic agents [147]. According to that, several randomized controlled clinical trials (Table 5) and meta-analyses have investigated the effect of lactoferrin administration on respiratory tract infections in both adult and pediatric populations, showing its overall effectiveness in reducing infection risk and improving clinical recovery, especially in infants and children [83,147,148]. In particular, when focusing on the pediatric population, the relevant positive results were consistent across different age ranges (infants, toddlers, and pre-school children) [147,149,150], while evidence in adults is still limited [83,151,152], possibly as a result of the different gastrointestinal metabolism of lactoferrin in pediatric and adult patients, as it is known that the different pH of the gastric environment led to a higher absorption of the intact molecule in infants [30,33].

Comprehensively, it has been hypothesized that lactoferrin administration could contribute to the reduction in respiratory symptoms not only by exerting antiviral and antimicrobial activities but also by acting as an immunomodulator [146,148], and particularly by regulating plasmacytoid dendritic cells activity [151].

Lactoferrin’s positive effects have also been observed in more critical conditions, such as acute lung injury and acute respiratory distress syndrome, two clinical conditions characterized by acute hypoxemic respiratory failure [146,153]. Animal models developed to study those conditions have highlighted that lactoferrin administration effectively reduced edema, cellular infiltration in bronchoalveolar lavage fluid (BALF), and pro-inflammatory cytokine (i.e., IL1β and IL6, and TNFα) production, while increasing IL10 levels in BALF [146,153]. From a mechanistic point of view, these diseases involve a de-regulation of several overlapping and interacting signaling pathways, and recent in vitro and in vivo studies revealed that the main targets of lactoferrin action in this context are represented by PPARγ and TLR4-NF-κB pathways, both representing key players in inflammatory responses and immune homeostasis maintenance [153,154].

Finally, several in vitro and in vivo studies have suggested a potential positive effect of lactoferrin administration for the treatment of other lung diseases, such as asthma, chronic obstructive pulmonary disease, and cystic fibrosis, but human studies are still lacking, highlighting the need of well-designed clinical trials to also demonstrate its therapeutic effectiveness (both in terms of main or additive treatment) in these clinical conditions as well as to provide clear clinical guidelines about doses and administration routes [3,146].

### 5.4. Coronavirus Disease 19 (COVID-19)

COVID-19 is currently recognized as the deadliest pandemic of the last century, characterized by very heterogeneous clinical manifestations, ranging from nearly asymptomatic flu-like conditions to severe symptoms leading to interstitial pneumonia, acute respiratory distress syndrome, multiorgan failure and, eventually, death [155,156]. Due to the lack of specific and effective drugs to fight the infection, especially during the first phases of the emergency, several bioactive molecules have been investigated to evaluate their preventive and/or therapeutic potential [157,158,159]. Among the bioactive compounds tested, bovine lactoferrin received great attention thanks to its tolerability and safety, along with its wide commercial availability [81,157,158,159].

The use of lactoferrin supplementation as an add-on to the standard-of-care therapy in treating COVID-19 has been hypothesized based on several in vitro evidences, supporting its ability to inhibit severe acute respiratory syndrome coronavirus 2 (SARS-CoV-2) replication [25,146,157] as well as its interaction with angiotensin-converting enzyme 2 (ACE2—the main entry receptor leading to host’s cells infection) [146,160], thus contrasting the very early infection stages. Furthermore, several in vitro and in vivo studies demonstrated lactoferrin’s ability to enhance immune responses [21,161] and to reduce the expression of key pro-inflammatory mediators of the COVID-19-associated cytokine storm, such as IL6 and TNFα [146,162,163], thus fostering the design of several clinical studies aimed at evaluating its effectiveness in modifying disease evolution.

To date, the available clinical studies led to mixed results [25,64,81], which are summarized in Table 6. Although the early studies on asymptomatic or mildly symptomatic patients reported lactoferrin’s supplementation ability to prevent SARS-CoV-2 infection [164], reduce the time to viral clearance, and improve clinical symptoms [165,166], the only three designed as controlled, randomized, clinical trials showed no protective effects against SARS-CoV-2 infection in healthcare professionals providing care to COVID-19 patients [167] as well as no additional benefits to the standard-of-care therapy in moderate-to-severe hospitalized patients [168,169].

In conclusion, this heterogeneous evidence could be explained by the different trials’ design, the type of bovine lactoferrin formulation, the timing and mode of therapeutic intervention, and the disease severity of the enrolled patients. Even if this limited evidence prevents the possibility of elaborating clear clinical guidelines, the proven lactoferrin antiviral activity against the emerging SARS-CoV-2 variants [64,170] fosters the design of clinical trials adequately powered to evaluate its effectiveness in improving disease evolution, possibly resulting in the identification of a preventive and/or supportive therapeutic agent, which could be a valuable intervention tool especially in resources-limited settings.

## 6. Lactoferrin as a Biomarker of Inflammatory Disorders

Lactoferrin is known to be part of neutrophils’ secondary granules content, from which is released by degranulation, determining the rapid increase in its plasmatic levels (0.4–2 µg/mL vs. up to 200 µg/mL) in response to inflammatory or infectious conditions [70,84]. Considering that activated neutrophils are one of the major drivers of inflammatory diseases, it is not surprising that neutrophil-derived lactoferrin levels have been considered a promising biomarker to evaluate disease severity. Nevertheless, the efficacy of lactoferrin as a biomarker for disease severity monitoring greatly varies among different diseases, showing good correlation with severity in some and low or null in others.

An example of good correlation between plasma lactoferrin levels and disease severity is represented by glaucoma, a chronic, progressive neurodegenerative condition which is among the major causes of blindness in the world. In an interesting cross-sectional study published in 2024, Wang and coworkers compared plasma lactoferrin levels in patients affected by glaucoma with different degrees of severity and healthy controls, showing good diagnostic accuracy of this biomarker in distinguishing between healthy subjects and patients affected by glaucoma, and between early and advanced stages of the disease [171].

Another disease in which neutrophil-derived lactoferrin has been evaluated as a potential biomarker for monitoring disease progression and severity is represented by rheumatoid arthritis, a progressive inflammatory autoimmune disease representing a public health challenge worldwide due to its continuously increasing incidence and prevalence [172]. Lactoferrin’s effectiveness in rheumatoid arthritis monitoring is more controversial. In previous studies, blood lactoferrin was reported to be a neutrophil activation marker rather than a disease activity marker [173,174]. Interestingly, in a very recent work, Delcheva and colleagues reported an inverse relationship between thioredoxin 1 and lactoferrin and rheumatoid factor but not with anti-cyclic citrullinated peptide antibodies and systemic disease activity. As the levels of these proteins were found to increase simultaneously in response to increased oxidative stress and disease activity (expressed as DAS28 value), the authors suggest a possible role of these proteins as a biomarker for detecting pathological changes and monitoring disease evolution [175].

As lactoferrin is known to be present in different exocrine secretions, it is not surprising that the studies about its effectiveness as a biomarker of disease also focused on other lactoferrin-rich fluids, such as tears. In particular, tears’ lactoferrin content has been proposed as a biomarker for Sjögren’s syndrome, an autoimmune disease characterized by inflammatory cell infiltration into exocrine glands such as the lacrimal ones [176]. Different studies reported a reduction in lactoferrin content in the tear film of patients suffering from Sjögren’s syndrome compared to healthy controls; nevertheless, as a reduction in tears’ lactoferrin content is a common finding also in dry eye disorders of other origin, its use as a diagnostic biomarker should be considered only in combination with other, more specific disease markers [176,177,178].

Finally, lactoferrin content has also been evaluated in stool as a biomarker for gastrointestinal disorders. In particular, fecal lactoferrin has proven to be a non-invasive and simple biomarker for differentiating between inflammatory bowel disease (IBD) and irritable bowel disease (IBS) [179]. As a matter of fact, lactoferrin in feces reflects the level of neutrophil translocation to the intestinal mucosa and thus represents a useful surrogate biomarker of inflammation. According to that, different studies reported good pooled sensitivity and specificity of fecal lactoferrin in differentiating IBD (both active and inactive) from IBS, a clinical condition with similar manifestations but different origin (functional vs. inflammatory) [179,180]. As fecal lactoferrin is recognized to reflect intestinal inflammatory status, its usefulness as a biomarker has been studied in the context of IBD. Considering that IBD could manifest as ulcerative colitis and Crohn’s disease, several authors have evaluated the effectiveness of fecal lactoferrin in differentiating the two conditions, showing that it is more effective in diagnosing and assessing ulcerative colitis rather than Crohn’s disease activity [181,182,183]. Furthermore, fecal lactoferrin has been evaluated as a biomarker of Crohn’s disease treatment response during anti-TNFα therapy (Infiximab). The comparison between pre- and post-intervention fecal lactoferrin levels has shown a good correlation with the endoscopic activity score, highlighting a significant decrease in this biomarker level in almost all patients reaching endoscopic remission [180,184]. Although fecal calprotectin has been reported to show good concordance with fecal lactoferrin in screening for IBD, there are some pros and cons to be considered when one of these biomarker evaluations is prescribed. On the one hand, fecal calprotectin is stable at room temperature for a longer time compared to fecal lactoferrin (~7 days vs. ~2 days), thus facilitating sample collection and delivery to the testing laboratory during follow-up visits. On the other hand, the existence of a single cutoff value for fecal lactoferrin makes easier the interpretation of a patient’s results over time compared to calprotectin, for which the cutoff value depends on the laboratory carrying out the test [185,186,187].

In conclusion, lactoferrin’s use as a disease-monitoring biomarker deserves attention, as it could represent an inexpensive and non-invasive technique for early screening, helping clinicians in identifying patients requiring more expensive and invasive diagnostic evaluations.

## 7. Conclusions

Lactoferrin is a multifunctional protein that has been extensively studied, especially for its usefulness as a nutritional supplement and therapeutic agent as well as a disease-monitoring biomarker. When specifically considering its therapeutic applications, in recent years, several studies have investigated novel sources of recombinant lactoferrin in order to improve the economical sustainability of the production process as well as novel forms of delivery (i.e., nanoparticles and liposomes in which lactoferrin has been used as the bioactive molecule or as a carrier for the targeted delivery of other drugs). On the other hand, considering its use as a nutritional supplement, lactoferrin has gained interest in the food industry, where it has been successfully used for different types of food fortification (i.e., milk, cheese, cream) and to improve the shelf life of different perishable compounds.

Novel sources of lactoferrin, especially those based on recombinant technologies, require safety and toxicity investigation to address the potential risk of allergic sensitization as well as the functional effectiveness of the resulting product, as it is known that one of the major drawbacks of recombinant lactoferrin is represented by the different patterns of glycosylation, according to the production source. On the other hand, lactoferrin therapeutic use highlights the need for well-designed clinical trials to ensure its safety as well as to confirm or disclaim its efficacy and to define clear clinical guidelines about the most effective administration schedule. Finally, the growing use of lactoferrin in the food industry deserves targeted investigation to define toxicity levels as well as to determine long-term effects of dietary supplementation.

## Figures and Tables

**Table 1:** Major bacterial targets of lactoferrin antimicrobial action.

Gram-Negative Bacteria	Reference	Gram-Positive Bacteria	Reference
*Aggregatibacter*	[3,45]	*Actinobacillus*	[45]
*Bacteriosides*	[45]	*Bacillus*	[45]
*Escherichia*	[3,24,46]	*Bifidobacterium*	[45]
*Enterobacter*	[45]	*Clostridium*	[3,45]
*Campylobacter*	[45]	*Corynebacterium*	[45]
*Chlamydophila*	[24]	*Enterococcus*	[45]
*Helicobacter*	[3,24,45]	*Lactobacillus*	[45]
*Klebsiella*	[24,45]	*Listeria*	[3,24,45]
*Legionella*	[24,45]	*Micrococcus*	[45]
*Mycobacterium*	[24]	*Staphylococcus*	[3,24,45]
*Porphyromonas*	[24]	*Streptococcus*	[3,24,46]
*Proteus*	[45]		
*Pseudomonas*	[3,45,46]		
*Salmonella*	[24,45,46]		
*Vibrio*	[24,45]		
*Yersinia*	[3,45]		

**Table 2:** Major viral targets of lactoferrin protective action.

Enveloped Viruses	Reference	Naked Viruses	Reference
*Chikungunya*	[7,47]	*Adenoviruses*	[3,45,46,50,62]
*Coronaviruses* (*SARS-CoV-1*, *SARS-CoV-2*)	[45,47,60]	*Echoviruses*	[3,45,46]
*Dengue*	[7]	*Enteroviruses*	[3,46]
*Feline herpes* (*FHV-1*)	[46,47]	*Human papilloma* (*HPV-5*, *HPV-16*)	[3,45]
*Hepatitis B* (*HBV*)	[3,45,46,50]	*Noroviruses*	[61]
*Hepatitis C* (*HCV*)	[3,45,46,47,50,62]	*Polioviruses*	[3,46,62]
*Hepatitis G* (*HGV*)	[45,46]	*Respiratory syncytial virus* (*RSV*)	[3,60]
*Herpes simplex* (*HSV-1*, *HSV-2*)	[3,7,45,47,50,62]	*Rotaviruses*	[3,45,46,50,60,62]
*Human cytomegalovirus* (*HCMV*)	[3,7,45,47,50,62]		
*Human immunodeficiency* (*HIV-1*, *HIV-2*)	[3,7,45,46,47,50,62]		
*Influenza A* (*H1N1*, *H3N2*, *H5N1*)	[45]		
*Hantaviruses*	[3,45,46]		
*Zika*	[7,47]		

**Table 3:** Summary of the main features of the clinical studies investigating lactoferrin effectiveness against *Helicobacter pylori* infection.

Country	Study Design	Participants	Intervention	Main Findings	Reference
Italy	Randomized, open-label, single-center clinical trial	Total of 74 consecutive patients (preliminary analysis—study designed to enroll 150 subjects). Total of 24 were allocated to group I, 26 were allocated to group II, and 24 were allocated to group III	Group I (400 mg/die bovine lactoferrin in addition to rabeprazole, clarithromycin, tinidazole) vs. group II (rabeprazole, clarithromycin, tinidazole) vs. group III (rabeprazole, clarithromycin, tinidazole). Intervention duration: 7 days for group I and II, 10 days for group III	Overall, 100% eradication rate with 7-day quadruple therapy (lactoferrin, rabeprazole, clarithromycin, tinidazole) vs. 76.9% with 7-day triple therapy and 70.8% with 10-day triple therapy	[126]
Italy	Prospective, randomized, open-label, multicenter clinical trial	Total of 133 consecutive adult patients with non-ulcer dyspepsia and *H. pylori* infection. Total of 65 were allocated to receive lactoferrin in addition to standard triple therapy and 68 were allocated to receive standard triple therapy alone	Amount of 400 mg/die lactoferrin in addition to standard triple therapy (esomeprazole, clarithromycin, amoxycillin) vs. standard triple therapy alone. Intervention duration: 7 days	No significant increase in *H. pylori* cure rate	[127]
Japan	Randomized, double-blind, placebo-controlled, single-center clinical trial	Total of 59 subjects (34 adults and 25 children). Total of 17 adults were allocated to receive lactoferrin and 17 were allocated to receive placebo. Total of 14 children were allocated to receive lactoferrin and 11 were allocated to receive placebo	Amount of 400 mg/die bovine lactoferrin vs. placebo (dextrin). Intervention duration: 12 weeks	Reduction in *H. pylori* colonization, as testified by a more than 50% decrease in the ^13^C-urea breath test value	[117]
Italy	Randomized, prospective, single center clinical trial	Total of 206 consecutive patients, where 105 were allocated to receive lactoferrin in addition to triple eradication therapy and 101 were allocated to receive triple eradication therapy alone	Amount of 400 mg/die bovine lactoferrin and probiotic formula in addition to triple eradication therapy (esomeprazole, clarithromycin, amoxicillin) vs. triple eradication therapy alone. Intervention duration: 7 days	Increase in the eradication rate and reduction in side effects of the triple therapy (nausea, diarrhea, metallic taste, abdominal pain, and glossitis)	[125]
Italy	Prospective therapeutic trial, based on two pilot studies carried out simultaneously	Total of 77 subjects (53 in one pilot study and 24 in the other); 53 were allocated to receive lactoferrin in addition to triple therapy and 24 were allocated to receive triple therapy alone	Amount of 300 mg/die bovine lactoferrin in addition to triple therapy (esomeprazole, amoxicillin, levofloxacin) vs. triple therapy alone. Intervention duration: 10 days	Increase in the successful eradication and cure rate	[115]
Egypt	Randomized, parallel, controlled, superiority, single-center clinical trial	Total of 400 patients; 100 were allocated to group I (analyzed 90), 100 were allocated to group II (analyzed 91), 100 were allocated to group III (analyzed 91), and 100 were allocated to group IV (93 analyzed)	Group I (400 mg/die bovine lactoferrin in addition to proton-pump-based triple therapy (esomeprazole, amoxicillin, clarithromycin)) vs. group II (400 mg/die bovine lactoferrin in addition to sequential therapy (esomeprazole, amoxicillin for 5 days, then esomeprazole, metronidazole, clarithromycin for 10 days)) vs. group III (proton-pump-based triple therapy alone) vs. group IV (sequential therapy alone). Intervention duration: 15 days	Increase in the successful eradication rate for both proton-pump-based triple therapy and sequential therapy when administered in combination with bovine lactoferrin	[124]

**Table 4:** Summary of the main features of the clinical studies investigating lactoferrin effectiveness against neonatal sepsis and necrotizing enterocolitis.

Country	Study Design	Participants	Intervention	Main Findings	Reference
Italy	Prospective, randomized, double-blind, placebo-controlled, multicenter clinical trial	Total of 472 very-low-birth-weight neonates; 153 were allocated to group I, 151 were allocated to group II, and 168 were allocated to receive placebo	Group I (100 mg/die bovine lactoferrin) vs. group II (100 mg/die bovine lactoferrin + 6 × 10^9^ CFU/die LGG) vs. placebo Intervention duration: 30 days (45 for neonates < 1000 g at birth)	Bovine lactoferrin, alone or combined with LGG, reduced the incidence of a first episode of late-onset sepsis in very-low-birth-weight neonates	[133]
Turkey	Prospective, randomized, double-blind, placebo-controlled, single-center clinical trial	Total of 50 very-low-birth-weight or premature (born before 32 weeks’ gestation) neonates; 25 were allocated to receive lactoferrin and 25 were allocated to receive placebo (22 analyzed)	Amount of 200 mg/die bovine lactoferrin vs. placebo. Intervention duration: until discharge	Bovine lactoferrin supplementation reduced nosocomial sepsis and increased regulatory T lymphocytes levels in very-low-birth-weight neonates	[134]
Italy and New Zealand	International, randomized, double-blind, placebo-controlled, multicenter clinical trial	Total of 743 very-low-birth- weight neonates; 247 were allocated to group I, 238 were allocated to group II, and 258 were allocated to receive placebo	Group I (100 mg/die bovine lactoferrin) vs. group II (100 mg/die bovine lactoferrin + 6 × 10^9^ CFU/die LGG) vs. placebo Intervention duration: 30 days (45 for neonates < 1000 g at birth)	Bovine lactoferrin, alone or combined with LGG, reduced the incidence of severe necrotizing enterocolitis (≥stage 2) and/or of death in very-low-birth-weight neonates	[135]
India	Randomized, double-blind, placebo-controlled, single-center clinical trial	Total of 132 neonates with birth weight < 2000 g; 65 were allocated to receive lactoferrin (63 analyzed) and 67 were allocated to placebo	Amount of 100 mg/die bovine lactoferrin for neonates between 1000 and 1249 g, 150 mg/die bovine lactoferrin for neonates between 1250 and 1499 g, 200 mg/die bovine lactoferrin for neonates between 1500 and 1749 g and 250 mg/die bovine lactoferrin for neonates between 1750 and 1999 g. Intervention duration: 28 days	Reduction in the incidence of the first episode of late-onset sepsis in low-birth-weight neonates	[136]
Peru	Pilot, randomized, double-blind, placebo-controlled, multicenter clinical trial	Total of 190 neonates with birth weight of 500–2500 g; 95 were allocated to receive lactoferrin (94 received the intervention) and 95 were allocated to placebo	Amount of 200 mg/kg/die bovine lactoferrin vs. placebo. Intervention duration: 4 weeks	No statistically significant reduction in late-onset sepsis, with confidence interval suggestive of an effect that justifies larger trials	[137]
United States of America	Randomized, blinded, placebo-controlled clinical trial	Total of 120 neonates with birth weight of 750–1500 g; 60 were allocated to receive lactoferrin and 60 were allocated to receive placebo	Amount of 300 mg/kg/die talactoferrin vs. placebo. Intervention duration: 28 days	Modulation of fecal microbiota and reduction in hospital-acquired infections in very-low-birth-weight infants	[138,139]
United Kingdom	Randomized, placebo-controlled multicenter clinical trial	Total of 2203 neonates born before 32 weeks’ gestation; 1099 were allocated to receive lactoferrin (1051 received the full intervention) and 1104 were allocated to receive placebo (1057 received the full treatment)	Amount of 150 mg/kg/die (maximum 300 mg/die) bovine lactoferrin vs. placebo (sucrose). Intervention duration: from the time at which the infant’s enteral feed volume was >12 mL/kg/die until 34 weeks’ postmenstrual age	No significant reduction in the risk of late-onset infections, mortality or other morbidity in very-preterm infants	[140]
Peru	Randomized, double-blind, placebo-controlled, multicenter clinical trial	Total of 414 neonates with birth weight of 500–2000 g; 209 were allocated to receive lactoferrin and 205 were allocated to receive placebo	Amount of 200 mg/kg/die bovine lactoferrin vs. placebo. Intervention duration: 8 weeks	No significant effects in decreasing the incidence of sepsis in infants with birth weight < 2000 g	[141]
Australia and New Zealand	International, pragmatic, randomized, double-blind, multicenter, superiority clinical trial	Total of 1542 neonates with birth weight < 1500 g and aged < 8 days; 771 were allocated to lactoferrin (770 analyzed) and 771 were allocated to no intervention group	Amount of 200 mg/kg bovine lactoferrin vs. no intervention. Intervention duration: until 34 weeks’ postmenstrual age (or for 2 weeks if longer) or until discharge (if it occurred first)	No significant effects on mortality, brain injury, stage II or III necrotizing enterocolitis, late-onset sepsis, and retinopathy treated before discharge	[142]

Abbreviations: LGG = lactobacillus rhamnosus GG; CFU = colony-forming units.

**Table 5:** Summary of the main features of the clinical studies investigating lactoferrin effectiveness against respiratory tract infections.

Country	Study Design	Participants	Intervention	Main Findings	Reference
United States of America	Pilot, randomized, double-blind, placebo-controlled, single-center clinical trial	Total of 79 infants aged between 0 and 4 weeks, born at ≥34 weeks of gestation and with a birth weight ≥ 2000 g. Of the 52 who completed the study period, 26 were allocated to the lactoferrin fortified formula group and 26 were allocated to the cow milk-based formula	Bovine lactoferrin fortified formula (850 mg/L) vs. cow milk-based formula (102 mg/L). Intervention duration: 12 months	Significant reduction in lower respiratory tract illnesses	[150]
China	Prospective, randomized, controlled, blinded, multicenter clinical trial	Total of 390 term infants aged between 4 and 6 months, exclusively breastfed but weaned; 130 were allocated to group I, 130 were allocated to group II and 130 were allocated to the control group	Group I (bovine lactoferrin fortified formula, containing 38 mg/100 g lactoferrin) vs. group II (lactoferrin-free formula) vs. control (exclusive breastfeeding). Intervention duration: 3 months	Reduced incidence of respiratory-related illnesses and fewer symptoms of running nose, cough, and wheezing in infants receiving bovine lactoferrin fortified formula or being exclusively breastfed	[149]
Japan	Randomized, double-blind, placebo-controlled, parallel-group, comparative, single-center clinical trial	Total of 310 adults; 103 were allocated to group I (95 analyzed), 103 were allocated to group II (96 analyzed), and 104 were allocated to receive placebo (99 analyzed)	Group I (200 mg/die bovine lactoferrin) vs. group II (600 mg/die bovine lactoferrin) vs. placebo. Intervention duration: 12 weeks	Dose-dependent attenuation of summer infectious diseases, including summer colds	[152]
Japan	Randomized, double-blind, placebo-controlled, single-center clinical trial	Total of 157 adult subjects; 78 were allocated to receive lactoferrin (68 analyzed) and 79 were allocated to receive placebo (77 analyzed)	Amount of 200 mg/die bovine lactoferrin vs. placebo. Intervention duration: 12 weeks	Reduction in the total score for respiratory and systemic symptoms and increase in CD86 and HLA-DR on plasmacytoid dendritic cells	[151]
Italy	Randomized, controlled clinical trial	Total of 50 children aged between 3 and 6 years, attending nursery/preschool, with a history of recurrent respiratory infections; 25 were allocated to receive lactoferrin and 25 were allocated to the control group	Amount of 400 mg/die bovine lactoferrin in addition to pharmacological treatment for ongoing infections (if needed) vs. no additional treatment to support pharmacological intervention for infections (if needed). Intervention duration: 4 months	Reduction in frequency and duration of respiratory infection episodes and reduction in corticosteroid use	[147]

Abbreviations: HLA-DR = human leukocyte antigen.

**Table 6:** Summary of the main features of the clinical studies investigating lactoferrin effectiveness against COVID-19.

Country	Study Design	Participants	Intervention	Main Findings	Reference
Spain	Prospective, observational study	Total of 75 COVID-19 adult patients in home-based isolation	Amount of 256–384 mg/die liposomal bovine lactoferrin nutritional syrup food supplement in addition to zinc solution or only liposomal bovine lactoferrin solution. Intervention duration: 10 days	Improvement of COVID-19 symptoms	[164]
Italy	Retrospective study	Total of 121 adult, asymptomatic, paucisymptomatic, and moderately symptomatic patients in home-based isolation; 82 were allocated to bovine lactoferrin and 39 were allocated to the bovine lactoferrin untreated group	Amount of 200 mg–1 g/die bovine lactoferrin (median dose: 400 mg/die in asymptomatic patients, 600 mg/die in paucisymptomatic patients, 1 g/die in moderate symptomatic patients) in addition to other medications (ibuprofen, paracetamol, azithromycin, heparin, and cortisone) depending on patient-specific conditions vs. other medications alone. Intervention duration: until the negativization	Reduction in the time to negativization	[166]
Italy	Randomized, parallel arm, open label, interventional clinical trial	Total of 92 adult, asymptomatic (25/92) and mild-to-moderate (67/92) COVID-19 hospitalized and in home-based isolation patients; 32 were allocated to group I, 32 were allocated to group II and 28 were allocated to group III. Total of 32 healthy volunteers represented the control group	Control group did not receive any treatment or placebo. Group I (1 g/die bovine lactoferrin for the oral administration and 16 mg/nostril/die bovine lactoferrin for the intranasal formulation) vs. group II (standard-of-care therapy) vs. group III (no COVID-19 treatment)	Reduction in the time to negativization and improvement of COVID-19 symptoms	[165]
Egypt	Randomized, single-center prospective, interventional clinical trial	Total of 54 adult patients hospitalized with mild-to-moderate COVID-19; 18 were allocated to the control group, 18 were allocated to group I, and 18 were allocated to group II	Control group (locally approved standard-of-care therapy) vs. group I (200 mg/die lactoferrin in addition to the standard-of-care therapy) vs. group II (400 mg/die lactoferrin in addition to the standard-of-care therapy). Intervention duration: 7 days	No additional benefits compared to the standard-of-care therapy	[169]
Italy	Randomized, multicenter, double blind, parallel arm, placebo-controlled clinical trial	Total of 218 adult patients hospitalized with moderate-to-severe COVID-19; 113 were allocated to receive lactoferrin (113 analyzed) and 105 were allocated to receive placebo	Amount of 800 mg/die bovine lactoferrin vs. placebo (corn starch) in combination with standard-of-care therapy. Intervention duration: 30 days	No additional benefits compared to the standard-of-care therapy	[168]
Peru	Randomized, double-blind, placebo-controlled, multicenter clinical trial	Total of 209 hospital workers; 104 were allocated to receive lactoferrin and 105 were allocated to receive placebo	Amount of 600 mg/die bovine lactoferrin vs. placebo (maltodextrin). Intervention duration: 90 days	No significant effect in preventing SARS-CoV-2 infection	[167]
